# Gut microbiome in hemodialysis patients treated with calcium acetate or treated with sucroferric oxyhydroxide: a pilot study

**DOI:** 10.1007/s11255-021-03091-3

**Published:** 2021-12-19

**Authors:** Ana Merino-Ribas, Ricardo Araujo, Ioana Bancu, Fredzzia Graterol, Andrea Vergara, Marc Noguera-Julian, Roger Paredes, Jordi Bonal, Benedita Sampaio-Maia

**Affiliations:** 1 Universitat Autònoma de Barcelona, Barcelona, Spain; 2 Nephrology Department, Hospital Universitari Germans Trias i Pujol, Badalona, Spain; 3 Nephrology Department, Hospital Universitari de Girona Doctor Josep Trueta, Avinguda de França S/N, 17007 Girona, Spain; 4 i3S—Instituto de Investigação e Inovação em Saúde, Universidade Do Porto, Porto, Portugal; 5 IrsiCaixa AIDS Research Institute, Hospital Universitari Germans Trias i Pujol, Badalona, Barcelona, Spain; 6 Faculty of Dental Medicine, University of Porto, Porto, Portugal

**Keywords:** Gut microbiome, Chronic kidney disease, Hemodialysis, Phosphate binders, Sucroferric oxyhydroxide, Calcium acetate

## Abstract

**Purpose:**

It has been proved that the gut microbiome is altered in patients with chronic kidney disease. This contributes to chronic inflammation and increases cardiovascular risk and mortality, especially in those undergoing hemodialysis. Phosphate binders may potentially induce changes in their microbiome. This trial aimed to compare the changes in the gut microbiome of hemodialysis patients treated with calcium acetate to those treated with sucroferric oxyhydroxide.

**Methods:**

Twelve hemodialysis patients were distributed to receive calcium acetate or sucroferric oxyhydroxide for 5 months. Blood samples (for biochemical analysis) and stool samples (for microbiome analysis) were collected at baseline, 4, 12, and 20 weeks after treatment initiation. Fecal DNA was extracted and a 16S rRNA sequencing library was constructed targeting the V3 and V4 hypervariable regions.

**Results:**

Regarding clinical variables and laboratory parameters, no statistically significant differences were observed between calcium acetate or sucroferric oxyhydroxide groups. When analyzing stool samples, we found that all patients were different (*p* = 0.001) among themselves and these differences were kept along the 20 weeks of treatment. The clustering analysis in microbial profiles grouped the samples of the same patient independently of the treatment followed and the stage of the treatment.

**Conclusion:**

These results suggest that a 5-month treatment with either calcium acetate or sucroferric oxyhydroxide did not modify baseline diversity or baseline bacterial composition in hemodialysis patients, also about the high-variability profiles of the gut microbiome found among these patients.

## Introduction

Chronic kidney disease (CKD) is a worldwide public health problem, with an increasing prevalence, a high economic burden, and elevated morbidity and mortality [1].

In CKD patients, cardiovascular pathology plays an important role. These patients present an increased risk of developing cardiovascular disease (CVD) and a cardiovascular mortality rate 30 times higher than the general population [2]. Besides the traditional cardiovascular risk factors that most of the time are more prevalent in patients affected by CKD than in the general population, the interconnection between CKD and CVD could be explained by the presence of bone and mineral disorders, hydration status, and inflammation that our patients develop.

Under normal conditions, inflammation is a protective and physiological response to various inimical stimuli. However, in several debilitating disorders, such as CKD, inflammation becomes harmful and persistent [3]. It is well known that CKD is accompanied by a persistent inflammatory status [4, 5]. Inflammation is likely the consequence of a multifactorial etiology and interacts with several factors that emerge when uremic toxins accumulate and has been described as a predictor of cardiovascular and total mortality [6]. Moreover, there is mounting evidence supporting the presence of intestinal barrier dysfunction and alterations in the gut microbiota composition in CKD, commonly referred to as gut dysbiosis [7–9]. This dysbiotic state concomitantly generates toxic by-products and contributes to the chronic status of oxidative stress and inflammation in these patients [10–12].

Several factors contribute to gut microbial dysbiosis in patients with advanced CKD. The accumulation of urea in body fluids and its diffusion to the gastrointestinal tract lead to the expansion of urease-possessing bacteria. Also, the hydrolysis of urea generates products that degrade the epithelial tight junction, thereby facilitating translocation of endotoxin and microbial fragments into the systemic circulation [7, 8, 10, 13–15]. Dietary recommendations in CKD including restricted intake of potassium, phosphate, sodium, and proteins result in a low intake of fermentable carbohydrates and this may lead to an expansion of proteolytic species and an increased generation of bacterial toxins [12, 13, 16]. Moreover, patients with CKD are commonly associated with other comorbid conditions, such as diabetes, autoimmune diseases, and hypertension. All these comorbidities result per se in gut microbiota alterations [17, 18].

A very important factor which favors dysbiosis are drugs. It is well known that patients with advanced CKD are usually poly-medicated. Iron supplementation or antibiotics, frequently used in our patients, have been demonstrated to alter the gut microbiome [19–21]. However, the effects on the gut microbiome of other widely used drugs in CKD patients remain unknown.

Most hemodialysis patients tend to present hyperphosphatemia and they need high doses of different types of phosphate binders to correct this condition*.* Phosphate binders can be classified as calcium and non-calcium-based phosphate binders. It has been described that both groups of phosphate-binding agents can potentially produce changes in the composition of the microbiome [22–25].

Recently, new non-calcium-based phosphate-binding agents have been approved for the treatment of hyperphosphatemia in hemodialysis subjects. Some of these new agents, such as sucroferric oxyhydroxide (SFO) and ferric citrate, hold iron in their compositions. It is believed that, given the critical role of iron in microbial growth and virulence, the large iron load administrated with these drugs, may alter gut microbiome composition [26, 27]. Nevertheless, there is still little evidence about the effects of these new phosphate binders on the gut microbiome [25].

Given the importance of the altered gut microbiome in CKD patients and its contribution to their inflammatory state, and the lack of information about the effects on the gut microbiome of these nowadays widely used drugs, we decided to monitor and compare the changes on the gut microbiome of patients undergoing hemodialysis taking SFO or calcium-based phosphate binder calcium acetate (CA).

## Materials and methods

### Recruitment

Twelve patients on hemodialysis in Hospital Universitari Germans Trias i Pujol were invited to participate in our study with a 5-month follow-up. All the subjects were recruited from the Hemodialysis Department of the Hospital Universitari Germans Trias i Pujol, in Badalona, Spain. All patients were aged above 18 years old and had been on hemodialysis for at least 1 year (4 h sessions, 3 sessions per week). This study was approved by the Clinical Research Ethics Committee of the Hospital Universitari Germans Trias i Pujol (PI-16-169, NCT5551048) and conformed to the principles outlined in the Declaration of Helsinki. All participants were recruited voluntarily after receiving detailed information on the study protocol. Written informed consent was obtained from all patients.

Exclusion criteria included inability to give informed consent, history of gastrointestinal disease, hospitalization, and antibiotics intake in the last 3 months.

Relevant clinical and demographic information was gathered for each individual at baseline. Clinical characteristics collected were: gender, age, CKD etiology, history of high blood pressure, diabetes mellitus, dyslipidemia, cardiovascular disease (peripheral vascular disease, ischemic cardiomyopathy or stroke), and cancer.

We also collected information regarding their vascular access, and their previous phosphate-binder treatment at the beginning of the study (nine received calcium acetate, one received calcium carbonate, and two were not previously treated for hyperphosphatemia).

We divided patients into two groups, and we changed their treatment for hyperphosphatemia: 5 patients were placed in CA group (4 continuing CA therapy and 1 patient changing from calcium carbonate therapy) and 7 were switched to SFO (5 changing from CA therapy and 2 starting phosphate-binding treatment).

### Sample collection

Fecal samples were collected from 12 hemodialysis patients receiving phosphate binders, 5 in the CA group and 7 in the SFO group. We collect also blood samples from the routine checks realized in our hemodialysis unit. The samples (blood and fecal samples) were collected in a 5-month follow-up: at baseline, 4, 12, and 20 weeks after treatment initiation.

In blood samples, we analyzed the following parameters: hemoglobin, ferritin, transferrin saturation index, calcium, phosphate, parathyroid hormone, C-reactive protein, sedimentation velocity, and albumin.

### DNA extraction, library construction, and sequencing

Fecal DNA was extracted by Powersoil DNA Isolation Kit MoBio, and a 16S rRNA sequencing library was constructed targeting the V3 and V4 hypervariable regions.

Sequencing was performed on a MiSeq platform (2 × 300). OTU table construction, taxonomic assignment, and descriptive and statistical analyses were performed using R version 3.4.2. and different packages (DADA 2, vega, ggplot, phyloseq) and the Greengenes rRNA database.

### Data and statistical analysis

Primer v7 (PRIMER-e, Auckland, New Zealand) was used for calculation of the diversity indices, similarity percentages (SIMPER) analysis, and multivariate analysis, mainly analysis of similarities (ANOSIM) one-way analysis and permutational multivariate analysis of variance (PERMANOVA; using squared root transformed data, Bray–Curtis similarities and 4999 permutations of residuals under a reduced model) used to test the significance of Beta-diversity. The percentage of OTU data per sample was used for these analyses, followed by squared root transformed data, resemblance matrices of similarity data types, using Bray–Curtis similarities, adding dummy value, and testing 4999 permutations. STAMP was used for analyzing taxonomic profiles among groups of samples and calculation of statistical differences [28].

For statistical treatment of the clinical data, the statistical analysis software Statistical Package for Social Sciences (SPSS) 26.0 for MAC OS was used. The categorical variables were described through relative frequencies (%) whereas continuous variables were described using mean ± standard deviation (SD). We applied when appropriate Chi-square independence test to analyze hypotheses regarding the categorical variables and Student’s t test concerning continuous variables. A level of 0.05 was considered significant.

## Results

The main clinical parameters were not different between patients assigned to CA or SFO groups at baseline (Table 1). We have observed that in the CA group, there was an increased prevalence, but not statistically significant, in history of arterial hypertension, dyslipidemia, peripheral vascular disease, stroke, ischemic cardiomyopathy than in the SFO group. The patients assigned to the SFO group presented a greater incidence of a catheter as vascular access, but also not statistically significant.

**Table 1:** Clinical characterization of patients undergoing calcium acetate (CA) or sucroferric oxyhydroxide (SFO) as phosphate-binding agent

Clinical parameter	CA	SFO
Age, years	66.8 ± 13.9	61.1 ± 16.7
Women, %	40.0%	42.9%
Arterial hypertension, %	100.0%	85.7%
Dyslipidaemia, %	60.0%	42.9%
Diabetes mellitus, %	40.0%	42.9%
Peripheral vascular disease, %	40.0%	14.3%
Stroke, %	40.0%	14.3%
Ischemic cardiomyopathy, %	40.0%	14.3%
Cancer, %	20.0%	28.6%
Catheter as a vascular access, %	60.0%	71.4%

Values are means ± SD or relative frequencies (%). No statistically differences found between CA vs SFO

At baseline, no patient was treated with SFO, some were treated with CA in both groups of treatment, and 2 patients in the SFO group have no phosphate-binding treatment. At this time point, we found no statistically significant differences regarding laboratory parameters, such as hemoglobin, ferritin, transferrin saturation index, calcium, phosphate, parathyroid hormone, C-reactive protein, sedimentation velocity, and albumin (Table 2). Collectively, we observed in the SFO group at baseline higher transferrin saturation indexes, and lower values of C-reactive protein than the CA group, but those differences were not statistically significant. We also noted in patients assigned to the CA group, an increased trend to hyperphosphatemia at baseline, but this was also not statistically significant.

**Table 2:** Laboratory clinical data of patients undergoing calcium acetate (CA) or sucroferric oxyhydroxide (SFO) as phosphate-binding agent

Laboratory parameter	CA	SFO
Ferritin, ng/ml		
Basal	1451.8 ± 1299.3	1185.1 ± 268.2
4 weeks	1670.4 ± 1326.9	1166.0 ± 187.2
12 weeks	1722.2 ± 1622.0	1056.8 ± 327.9
20 weeks	1691 ± 1557.1	1149.8 ± 360.0
Transferrin saturation, %		
Basal	29 ± 8.9	53.1 ± 29.4
4 weeks	43.4 ± 19.7	46.7 ± 28.9
12 weeks	38.8 ± 19.3	41.4 ± 18.6
20 weeks	32.4 ± 14.0	42.0 ± 15.6
Calcium, mg/dl		
Basal	9.34 ± 0.3	9.1 ± 0.3
4 weeks	9.24 ± 0.3	8.9 ± 0.4
12 weeks	9.02 ± 0.5	8.7 ± 0.7
20 weeks	10.06 ± 0.7	8.9 ± 0.5*
Phosphate, mg/dl		
Basal	5.16 ± 2.1	4.4 ± 2.2
4 weeks	4.88 ± 1.4	4.4 ± 1.8
12 weeks	4.42 ± 1.7	5.5 ± 2.7
20 weeks	3.26 ± 0.8	4.7 ± 2.9
Parathormone, pg/ml		
Basal	242.1 ± 182.7	216.9 ± 259.2
4 weeks	309.4 ± 211.2	244.1 ± 267.7
12 weeks	327.1 ± 196.9	254.5 ± 380.9
20 weeks	181.5 ± 136.8	134.4 ± 108.8
C-reactive protein, mg/ml		
Basal	16.9 ± 20.6	4.2 ± 2.6
4 weeks	12.02 ± 6.0	5.6 ± 7.0
12 weeks	12.54 ± 7.3	4.4 ± 3.9
20 weeks	7.78 ± 6.3	4.2 ± 1.7
Sedimentation velocity, mm		
Basal	51.4 ± 24.2	45.3 ± 19.1
4 weeks	46.8 ± 24.2	39.2 ± 18.3
12 weeks	61.6 ± 26.9	26.4 ± 17.6*
20 weeks	47.6 ± 7.2	38.4 ± 19.4
Albumin, g/l		
Basal	39.04 ± 2.2	35.7 ± 3.0
4 weeks	37.54 ± 1.6	35.4 ± 2.4
12 weeks	37 ± 3.7	33.9 ± 4.0
20 weeks	39.5 ± 1.8	32.8 ± 2.3*
Hemoglobin, g/dl		
Basal	11.26 ± 0.9	10.9 ± 1.4
4 weeks	11.42 ± 0.8	10.1 ± 2.9
12 weeks	10.14 ± 1.3	11.8 ± 1.3
20 weeks	10.55 ± 0.8	10.7 ± 1.2

Values are means ± SD. *Values in SFO are significantly different from CA

In Table 2, we present the evolution of the laboratory parameters over the different time points. We found that in the CA group, the 20-week calcium was higher than in the SFO group with statistical significance (*p* = 0.02). Sedimentation velocity was increased in the CA group at week 12 of treatment when compared with the SFO group, with statistical significance (*p* = 0.04). Also, a statistically significant lower albumin was observed in the SFO group at 20-week treatment when we compare it with the CA group (*p* < 0.01). The ferritin levels in both groups at baseline and after 20 weeks of treatment were high in the two groups, and both groups get normal levels of phosphate at 20 week of treatment, with no statistically significant differences. The levels of transferrin saturation indexes, parathormone, C-reactive protein, sedimentation velocity, and albumin, at 20 weeks of treatment were similar in both groups.

The samples of all time points (baseline, week 4, week 12, and week 20) were collected in eight out of the total twelve individuals, in a total of 38 stool and blood samples. From the initial set of 12 patients, patient 7 (SFO group) dropped out because he was derived to another hospital due to clinical reasons and we could no longer monitor all the variables relevant for the study, patient 3 (SFO group) received a kidney transplant before the collection of 20-week samples, patient 9 (SFO group) died before the collection of 12-week samples, and we only could get good-quality samples for gut microbiome from week 12 and week 20 on patient 8 (SFO group).

The set of 38 fecal samples showed over 2 million reads, then classified using the Greengenes database. A high number of ASVs (33,734) were found among the tested samples and classified as belonging to the kingdom Bacteria. Shannon diversity was measured in each sample and the group of 38 samples showed values for Shannon diversity ranging from 6.2 to 7.7.

**Fig. 1:**
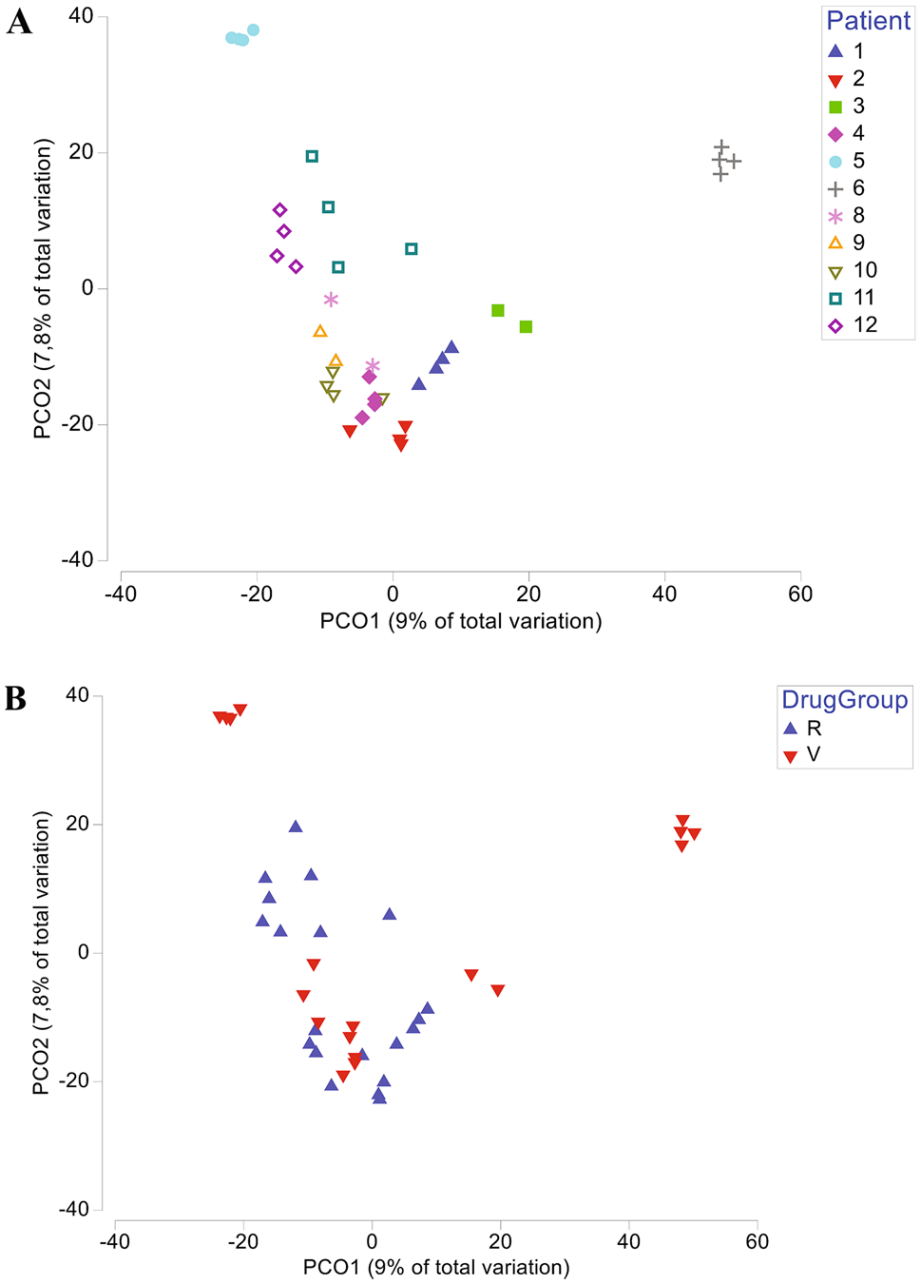
**A** Principal Co-ordinate Analysis (PCO) of the microbiome profiles for multiple patients. **B** Principal Co-ordinate Analysis (PCO) of the microbiome profiles for drug treatments (calcium acetate versus sucroferric oxyhydroxide)

Interestingly, we found that all patients were very different among themselves (*p* = 0.001) when comparing one patient with another patient at baseline (Fig. 1A). These differences among the patients were kept along the 20 weeks of treatment; there were no significant differences (*p* > 0.05) when the samples were grouped by week of treatment (baseline, 4, 12, or 20 weeks). It is important to note that the gut microbiome was found stable throughout the 20 weeks of study in patients that were on CA before the study and maintained that therapeutic within the study protocol, and also in patients who changed phosphate-binding therapeutics (from no treatment, CA or calcium carbonate to CA or SFO).

When we compared the microbial profiles of the patients treated with CA versus SFO considering all time points, we found statistical differences (Fig. 1B); and these differences were confirmed by ANOSIM (*p* = 0.002) and PERMANOVA (*p* = 0.001). This statistical analysis was done independently of the differences observed at baseline.

**Fig. 2:**
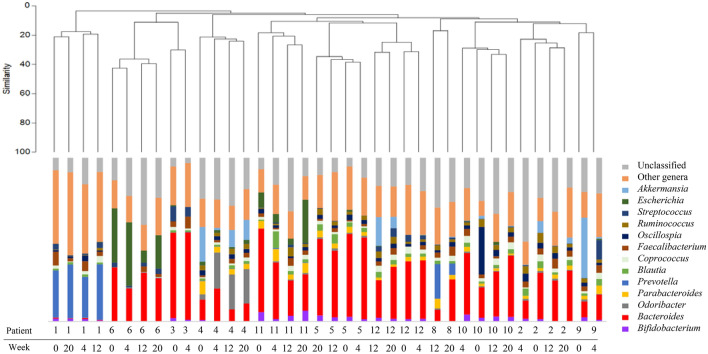
Clustering analysis and microbiome profiles (at genus level) for the samples considered in this study

The bacterial communities were studied and Bacteroidetes and Firmicutes were the most common phyla found in the fecal samples, followed by Proteobacteria, Actinobacteria, and Verrucomicrobia. Looking for more specific compositional differences, we compared multiple taxonomical levels among these samples. When analyzing the bacterial composition at the genus level, *Bacteroides* was the most prevalent in both groups of patients, independently if they were treated with either CA or SFO (Fig. 2). The microbial profiles were very distinct among patients and, once again, the clustering analysis grouped the samples of the same patient independently of the treatment followed and the stage of the treatment (baseline, 4, 12, or 20 weeks).

When we consider all the time points (all patients and all weeks), it was possible to find statistical differences (*p* < 0.05) for the microbial communities when comparing the samples for multiple variables, including gender, ischemic cardiomyopathy, the use of a catheter as vascular access, or age (the patients were organized in three groups: under 45, range 61–69, above 71). Such statistical differences could not be observed when each treatment stage (baseline, 4, 12, or 20 weeks) was considered separately; therefore, no differences were observed for the variables gender, age, ischemic cardiomyopathy, catheter use, and drug treatments (CA versus SFO).

## Discussion

This is the first study comparing the changes in the gut microbiome of hemodialysis patients taking CA versus SFO. In our study, there were no consistent differences in the bacterial composition of the gut microbiome between these different phosphate-binding agents. Although we found different microbiome profiles when both groups of treatment were compared, this different profile was present already at baseline, and long-term treatment did not modify this diversity in any of the two groups. So, no significant changes were observed over different time points (baseline, week 4, week 12, week 20) in hemodialysis subjects gut microbiome treated with CA versus those treated with SFO.

The gut microbiome of our patients is in accordance with previous reports, dominated by Bacteroidetes and Firmicutes phyla, being Actinobacteria, Proteobacteria, and Verrucomicrobia in the second line of colonization [29]. As previously extensively discussed, CKD patients have numerous intrinsic factors that promote gut dysbiosis besides the pharmacological therapies, namely reduced colonic transit, altered digestive capacity, metabolic acidosis, intestinal wall edema, and one of the most important, the high intestinal availability of uremic toxins. In comparison to healthy controls, patients undergoing hemodialysis present an increased Bacteroidetes abundance [30], also corroborating with the results of our study.

There is little evidence about the effect of phosphate binders on the gut microbiome [25]. Studies assessing the effects of calcium-based phosphate binders, including CA, on the CKD patients gut microbiome, analyzing fecal samples, are lacking [31]. Trautvetter et al. [32], observed an increase of fecal total short-chain fatty acids and a higher relative abundance of the genus *Clostridium* XVIII in healthy individuals taking calcium carbonate. Navarro-Gonzalez et al. [22], analyzed hemodialysis patients serum samples taken either the non-calcium-based phosphate binder sevelamer or the calcium-based phosphate binder CA and concluded that treatment with sevelamer was associated with a significant decrease in high-sensitive C-reactive protein, IL-6, serum endotoxin, and soluble CD14 concentrations independent predictors of mortality in hemodialysis patients.

SFO is an iron-based phosphate binder, and data suggested that the iron contained in the compound may switch gut microbiota because some gut bacteria use iron to increase relative abundance [26, 33, 34]. Moreover, it has been shown that an increase in the amount of iron reaching the colon may promote virulence of some pathogenic bacteria and a pro-inflammatory environment [20, 35]. But despite this evidence, our study shows that SFO treatment in hemodialysis patients does not seem to modify the gut microbiome, nor CA treatment.

Ling Lau et al. [27], compared fecal microbiome and uremic toxins in serum samples between CKD rats (who underwent 5/6 nephrectomy) and normal rats, randomly assigned to a regular diet or a diet containing 4% ferric citrate for 6 weeks. They observed that CKD rats had lower relative abundances of some Firmicutes and *Lactobacillus* and a lower gut microbial diversity compared to normal rats, but they also described that ferric citrate treatment in CKD rats increased bacterial diversity almost to levels observed in control rats and that this treatment did not increase uremic toxins. In a recent study, Wu et al. [36], compared hemodialysis patients gut microbiome treated with either calcium carbonate or ferric citrate. They observed a significantly increased microbial diversity in the group treated with ferric citrate, with an increased abundance of Bacteroidetes and a decreased abundance of Firmicutes.

To our knowledge, there is only one study performed in humans regarding SFO effect on the gut microbiome. Iguchi et al. [37], compared 3 months’ changes in the gut microbiome and uremic toxins of hemodialysis patients treated with either SFO versus no treatment for hyperphosphatemia. They also found no changes in the gut microbiome in patients treated with SFO throughout time. So, our study confirms this long-term stability of the gut microbiome in hemodialysis patients treated with SFO for 5 months.

Another important point to discuss is that in our study, we observed differences in hemodialysis patients gut microbiome compared by age or gender, but we have not found differences when we compare them by group of treatment (CA versus SFO). In accordance, some alterations have been demonstrated in the gut microbiome by aging [38]. Elderly patients, especially those with high frailty scores, present relative proportions of Bacteroidetes predominating, less microbial diversity, and decreases in *Bifidobacteria*, *Bacteroides*/*Prevotella, Lactobacillus,* and *Clostridium* cluster IV, when compared with young individuals, which present more microbial diversity and higher proportions of Firmicutes, among others [39, 40]. There is also mounting evidence supporting that there are alterations in the gut microbiome if comparing women and men [41, 42]. In our study, some differences were observed in the gut microbiome according to gender and age, but the differences found among each patient were much more pronounced.

Regarding laboratory findings, as expected, patients treated with the calcium-based phosphate binder CA presented higher calcium levels than those treated with SFO. We observed, although not statistically significant, increased levels of inflammatory parameters, such as sedimentation velocity, C-reactive protein, and ferritin in the CA group when compared with the SFO group; such pleiotropic effect on diminishing inflammation was described for some phosphate binders other than calcium-based binders [43].

It is essential to consider that our study presents some limitations. On the one hand, the size of the patient sample is small, so it is difficult to draw solid conclusions, especially on the effects of the clinical and biochemical variables analyzed. To validate our results, a larger study, with an increased number of patients is needed. On the other hand, our patients display different backgrounds, with distinctive comorbidities which can influence the gut microbiome. So, our study alerts about the high variability of profiles found on the gut microbiome of patients receiving phosphate binders. Such differences limit any additional conclusions and differences found among the patients receiving different phosphate binders.

As we reported, to search if a specific clinical variable could influence this differentiated microbiome profile, we analyzed the main clinical parameters at baseline of our patients and we found not statistically significant differences between both groups of treatment. Patients 5 and 6 were a little bit out of order and it can be stated that patient 5 received vancomycin and tobramycin for 3 weeks, while patient 6 presented chronic diarrhea with repeatedly negative cultures and a possible wasting syndrome associated.

In our study, we observed no changes in the gut microbiome of our hemodialysis patients after 20 weeks of treatment, independently of the phosphate binder. For the moment, when choosing a phosphate binder, we should rely on their power on the reduction of serum phosphate, the pill burden, the association to the vascular calcification progress, the adverse events, or the gastrointestinal tolerance [44–47]; although the influence of these phosphate binders on gut microbiome was expected, and still remains possible, for now, there is no evidence that this aspect should influence our approach when treating hyperphosphatemia.

In conclusion, our study observed that 5-month treatment with either CA or SFO did not modify baseline diversity nor baseline bacterial composition in hemodialysis patients, but alerts about the high variability of profiles found on the gut microbiome of CKD patients.

## Data Availability

The datasets used and/or analyzed during the current study are available from the corresponding author on reasonable request.
